# Barriers and facilitators to kangaroo mother care implementation in Cote d’Ivoire: a qualitative study

**DOI:** 10.1186/s12913-021-07086-9

**Published:** 2021-11-09

**Authors:** Kadidiatou Raïssa Kourouma, Marie Laurette Agbré-Yacé, Daouda Doukouré, Lassina Cissé, Chantière Some-Méazieu, Joseph Ouattara, Akoua Tano-Kamelan, Virginie Konan Kouakou

**Affiliations:** 1 Cellule de Recherche en Santé de la Reproduction, Institut National de Santé Publique, Abidjan, Côte d’Ivoire; 2 Service de Pédiatrie, Centre Hospitalier Universitaire de Treichville, Abidjan, Côte d’Ivoire; 3 United Nation Children’s fund (UNICEF) Cote d’Ivoire, Abidjan, Côte d’Ivoire

**Keywords:** Health system, Kangaroo mother care, Mothers, Healthcare providers, Barriers, Facilitators

## Abstract

**Background:**

Kangaroo Mother Care (KMC) is a high impact, low technology and cost-effective intervention for the care of preterm and low birth weight newborn. Cote d’Ivoire adopted the intervention and opened the first KMC unit in 2019. This study aimed to assess barriers and facilitators of KMC implementation in Cote d’Ivoire, a year after its introduction, as well as proposed solutions for improving KMC implementation in the country.

**Method:**

This was a qualitative study, using semi-structured interviews, carried out in September 2020 in the first KMC unit opened at the Teaching Hospital of Treichville. The study involved healthcare providers providing KMC and mothers of newborn who were receiving or received KMC at the unit. A thematic analysis was performed using both inductive and deductive (Consolidated Framework for Implementation Research-driven) approaches. NVivo 12 was used to assist with coding.

**Results:**

A total of 44 semi-structured interviews were conducted, 12 with healthcare providers and 32 with mothers. The barriers identified were lack of supplies, insufficiency of human resources, lack of space for admission, lack of home visits, lack of food for mothers, lack of collaboration between health services involved in newborn care, increased workload, the beliefs of carrying the baby on the chest, father’s resistance, low rate of exclusive breastfeeding, lack of community awareness. Facilitators identified were training of healthcare providers, strong leadership, the low cost of KMC, healthcare providers’ perceived value of KMC, mothers−healthcare providers’ relationship, mothers’ adherence to KMC and the capacity of the KMC unit to network with external organizations. The proposed solutions for improving KMC implementation were volunteer staff motivation, intensifying education and counselling of mothers and families, the recruitment of a psychologist and the involvement of all stakeholders.

**Conclusion:**

Our study highlighted the challenges to implement KMC in Cote d’Ivoire with unique and specific barriers to implementation. We recommend to researchers and decision makers to respectively design strategies and adopt intervention that specifically address these barriers and facilitators to a better uptake of KMC. Decision makers should also take into account the proposed solutions for a better implementation and scaling up of KMC.

**Supplementary Information:**

The online version contains supplementary material available at 10.1186/s12913-021-07086-9.

## Background

Health systems at all levels of development are under pressure to provide high quality of care based on the best available evidence. As available evidence, the use of Kangaroo Mother Care (KMC) is recommended by the World Health Organization (WHO) as a high impact, low technology and cost-effective intervention for the care of preterm and low birth weight (LBW) newborns who are at high risk of neonatal mortality and morbidity [1–4]. KMC comprises a set of care practices, including early continuous and extended skin-to-skin contact between infant and caregiver; and exclusive breastfeeding. KMC practice is often associated with early discharge from hospital and necessary support for caregiver and infant at home [4, 5]. WHO recommends the continuation of KMC until the newborn’s gestational age reaches term (gestational age around 40 weeks) or the weight reaches 2500 g [4].

KMC is a proven intervention in improving the survival and health of high risk newborn in low and middle income countries (LMICs) especially those in sub-Saharan Africa and parts of Asia, where the majority of neonatal deaths occurs and incubators are not widely available [6]. Indeed, the estimated global preterm birth rate for 2014 was 10.6%, equating to an estimated 14.84 million live preterm births; with 81.1% of these preterm births that occurred in Asia and sub-Saharan Africa [7].

KMC is epitomized by the Every Newborn Action Plan (ENAP) [8], and adopted in many countries such as India, Indonesia, Ghana, Uganda, South Africa, Malawi, Papua New Guinea, Mali, Cote d’Ivoire, which have made it a priority [9–18].

Cote d’Ivoire, a francophone West African country, operationalized KMC in 2019 following the adoption of the ENAP 2018–2020 [18]. In 2018, the country counted 25,195,540 inhabitants with a number of expected birth around 880,740 [19]. Moreover, the national percentage of LBW and premature decreased from 11.5% in 2017 to 10.8% in 2018, but remained slightly above the 2018 national goal (< 10%) [19]. The neonatal mortality rate accounted for more than half (56%) of all infant deaths recorded in Cote d’Ivoire. Although a clear reduction has begun, the mortality of newborn aged 0–28 days remained high at 33‰ [18]. The leading causes of death were preterm birth complications (30%), intrapartum related events (28%), sepsis/tetanus (21%), congenital abnormalities (7%), pneumonia (7%), diarrhea (1%), other conditions (7%) [20]. Thus, the Ministry of Health with the financial and technical support of UNICEF through the French Muskoka Fund, opened in February 2019, the first KMC unit in the Teaching Hospital of Treichville (CHUT) with the objective to scale up this intervention. However, to adequately implement and effectively scale up KMC intervention, it is fundamental to understand factors that may influence the intervention. Indeed, as each context setting presents organizational, professional, maternal and cultural particularities, identification of facilitators and barriers is critical to improve the implementation of clinical practice strategies [21, 22].

Despite many studies published on barriers and drivers of implementing KMC [5, 9–14, 17, 22–41], we assumed that Cote d’Ivoire may have some unique barriers and facilitators to the practice of KMC due to the country specificities, notably in terms of policies, resources and traditional culture. Furthermore, to the best of our knowledge, there are very few studies conducted in francophone West Africa and we are not aware of any prior published studies in Cote d’Ivoire exploring these barriers and facilitators. Therefore, we carried out a qualitative study to investigate barriers and facilitators of KMC implementation and proposed solutions to improve KMC implementation.

## Conceptual framework

This study is part of a larger project that aimed to document the implementation process of KMC in Cote d’Ivoire. The whole project was guided by the Consolidated Framework for Implementation Research (CFIR) as a conceptual framework. We chose the CFIR for its flexibility to assess implementation process as well as barriers and facilitators [42]. The CFIR that is adapted from other implementation theories and frameworks, is composed of 39 constructs that address different aspects of intervention implementation. These 39 constructs are organized into five major domains: intervention characteristics, outer setting, inner setting; characteristics of individuals and process. The use of the CFIR provides a comprehensive framework to systematically identify factors that may emerge in various, multi-level contexts to influence implementation [43]. This conceptual framework can help to produce findings to inform stakeholders on improvements to the intervention and its implementation. Additionally, we used the CFIR to facilitate the comparison with other implementation programs and studies.

## Method

The present study is part of a larger project that aimed to document the implementation process of KMC in Cote d’Ivoire throughout process evaluation (including fidelity), acceptability, sustainability, scaling up, barriers and facilitators. In the current study, we collected data using semi-structured interviews to identify barriers and facilitators of KMC implementation as well as proposed solutions to improve its implementation.

### Study setting

This study was conducted in September 2020 in the KMC unit of the CHUT created in February 2019 with the technical support of UNICEF through the French Muskoka fund. Prior to its opening, some healthcare providers of the neonatology intensive care unit (NICU) received training on KMC and perinatal death review at the Kalafong Hospital of Pretoria (South Africa) in order to become a national pool of trainers for cascade training. The rehabilitation of the oral rehydration center was carried out to create the KMC unit. The unit is part of the (NICU), however the KMC unit is located on the ground floor and the NICU on the second floor. The KMC unit is composed of the pediatrician’s and midwives’ offices, one training room, one living room equipped with a television for educational sessions, a large room with a capacity of nine beds with armchairs for mothers, two bathrooms for mothers and healthcare providers, and one dining room adjacent to a small kitchen for the mothers to cook. In the unit, only continuous KMC is provided, however in some case intermittent KMC is initiated in NICU before the dyad leaves for the KMC unit. The medical staff working at the unit is composed of 01 pediatrician, 02 midwives, 02 nurse aids, 01 childcare worker and 02 volunteers. All the healthcare providers working at the KMC unit have received a training in KMC including a short module on breastfeeding management and counselling. The midwives, the nurse aids and the childcare are counsellors in breastfeeding but need refresher trainings. To strengthen their capacities in breastfeeding management and counselling, the Programme National de Nutrition (PNN) was approached to collaborate with the unit. Upon their admission in the unit, healthcare providers help mothers to settle in and prepare (e.g, positioning the child, etc.) for skin-to-skin contact and child feeding.

Throughout their stay, mothers are counselled on infant feeding and trained to recognize danger signs in their newborns. Then, near the end of their stay, during the preparation to discharge, mothers are assessed on their ability to implement KMC at home and are emotionally prepared. Families are also advised to make arrangements within the household for mothers to continue KMC.

### Study design

Our aim was more to identify themes than interpret perspectives, therefore we employed qualitative descriptive methodology with thematic analysis to study the question what are the facilitators and barriers to KMC implementation as well as proposed solutions to improve implementation? To collect data for the study, we convened to use semi-structured interviews.

### Participants and sampling

The study population was composed of two main categories of participants: mothers and healthcare providers.

Concerning mothers, the sample comprised the mothers admitted with their child who were receiving KMC and those discharged who received KMC. We did an iterative sampling, moving back and forth between selecting mothers for data collection and engaging in a preliminary analysis of the interviews performed. The idea was that what emerges from data analysis would shape subsequent sampling decisions. We used the principle of saturation to end the interviews with mothers. The process of iterative sampling and analysis continued until we reached saturation and no new information and code were emerging any longer from data analysis.

As for healthcare providers, we made a purposive sampling in order to select the healthcare providers according to their capacities to provide richly-textured information, relevant to the research question under investigation. Healthcare providers were recruited if: they worked at the KMC unit or a general hospital that belongs to the perinatal network and refer newborn at the KMC unit and if they had received training on KMC. Thus, the sample included the healthcare providers working at the KMC unit of the CHUT (08) and the healthcare providers in charge of KMC in the following four general hospitals (04) that belong to the perinatal network: Marcory, Koumassi, Port-Bouët, and Treichville. These general hospitals refer preterm and LBW newborn to KMC unit. Each healthcare provider was an informant for each general hospital.

In the first draft of the protocol, it was planned to conduct focus groups in the community with mothers, husbands and other relatives that participate in the newborn care. However, due to COVID-19 pandemic restriction such as lockdown, we decided to carry out the study solely in the unit. Moreover, healthcare providers and mothers are the main actors of KMC implementation and were more likely to be present at the unit.

### Data collection

Two interview guides, one for each target population, were designed by the research team around the following topics: perception of KMC, practice of KMC, barriers and facilitators of KMC implementation and proposed solutions to improve implementation.

Data were collected by two data collectors with a master’s degree in sociology. They received one-day training on general survey procedures, including health protocol for COVID-19 prevention, the content of the interview guides [see Additional file 1] and a short module on KMC. This one-day training was carried out to ensure comprehension of questions by the data collectors and that they would conduct interviews and collect data in an identical manner. The interview guides were initially piloted on a small sample to ensure that questions were relevant and easily understood by the interviewees. Only small changes were made after the pilot-test, such as a minor rephrasing of questions.

Mothers who came for visits were called two days before data collection to confirm if they would come to the KMC unit. During data collection, mothers were invited by the data collectors to participate in the interviews in a room located in the pediatric ward outside the KMC unit so that they would be more comfortable answering questions. As for healthcare providers, they were invited to an individual one to one interview at the KMC unit. Prior to interviews, all participants were asked to provide written consent after a further opportunity to have their questions answered. All participants agreed to have their interview recorded. Interviews lasted on average 40 min (range 35–45 min) and were conducted in French. However, we tried to adapt the level of French to the participant’s education level.

Regarding mothers’ interviews, saturation was reached after the 32nd interview. To confirm that data saturation has been reached, we chose to carry out two additional interviews. Thus, a total of 42 mothers were approached, out of them, 10 did not agree to participate mainly because of lack of time. Thirty-four participated in the study, including the two additional interviews to confirm data saturation (these two additional interviews were not included in the analysis). As for healthcare providers’ interviews, all of them participated in the study.

We immediately conducted debriefing sessions with data collectors after each data collection day to have an overview of the content of the collected data, to improve the data collector skills, to make adjustments in case of challenges or unforeseen events and also to strengthen the quality and trustworthiness of data in real time.

### COVID-19 precautions

Regarding prevention of COVID-19 infection, the following health protocol has been implemented in the unit:

- At the KMC unit level: only five beds were left open to maintain physical distancing between mothers, in addition to the other measures to prevent COVID-19 infection, renewal of awareness and hand washing demonstration posters, compliance with barrier measures
- At the mother’s level: free distribution of 40 face masks to each mother per month, regular hand washing or use of hand sanitizer, respect of physical distancing with the other parents and visitors, reception of visitors in the courtyard, restriction of visits to the strict minimum and when necessary.

At our level we also took measures for the prevention of COVID-19 infection during fieldwork. Indeed, because of the risks associated with face-to-face interviews, data collectors were PCR-tested two days before fieldwork launch and declared negative. As for the room allocated for the interview, we ensured proper ventilation with outside air in order to prevent COVID-19 infection. It was mandatory to use masks for both data collectors and participants. Data collectors and participants had to wash their hands or use hand sanitizer before and after each interview. Data collectors were required to observe physical distancing (one meter) at all times. This included not shaking hands or having any physical contact with any participants. Once fieldwork was finalized, all data collectors and supervisors were PCR-tested and declared negative.

### Data analysis

All interviews were transcribed verbatim in Microsoft Word from audio-recordings by the first (KRK) and the third author (DD). They checked for accuracy and quality the first three interviews in the presence of the data collectors as they were the most familiar with the content of the interviews and could quickly identify any problems with the transcripts. Then, they performed a spot-checking by taking a subset of the remaining transcripts (six transcripts selected randomly per transcribers) and listened to the entire taped interviews of those transcripts while checking the transcripts in the presence of the data collectors. The resulting transcripts were de-identified, seen only by study team members, and housed securely in an online storage service. Thematic analysis was carried out using both inductive and deductive approaches. NVivo 12 software was used to assist with coding. Data were coded in two phases. In the first phase all data were organized in the software program NVivo 12 and coded using the grounded theory [44]. Both authors (KRK and DD) independently read each transcript several times to identify emerging themes relevant to the research question and coded the quotes that represented each theme. When new themes emerged, the researchers returned to the previous transcripts to re-read and recode them, if necessary. The transcript was coded line by line and each idea was given a name or word summarizing the main idea or concept. The process of developing was iterative and involved numerous conversations among the coders and other members of the research team. They compared their codes in regular meetings to review and refine their codes, and to discuss emerging themes. Ten interviews were inter-coded before agreement reached and the percent agreement was 72% (number of codes that all two coders agreed on divided by the number of total coded sections). The final categorization was approved by all the research team members during a meeting. Then, three of the authors (KRK, DD, and AYML) reviewed the themes and sub-themes. Any discrepancies were resolved throughout several discussions and meetings of the research team. In the second phase of analysis based on a deductive approach, the CFIR was used to frame the results in a comprehensive and systematic manner by applying key domains that are considered most salient in program implementation [42]. KRK and DD mapped each theme to relevant constructs within the CFIR model by considering how the theme was related to intervention characteristics, inner setting, outer setting, characteristics of individual and process. We performed triangulation of data sources.

### Data rigor and trustworthiness

Several strategies were used to ensure data rigor and trustworthiness. Transferability was established by providing a thick description of the study settings. To establish confirmability, the records and the field note were kept in order to be available for re-analysis. Dependability was ensured by describing the research process in detail, piloting the interview guides and ensuring comparable data collection procedure between data collectors, conducting debriefing sessions. Credibility was achieved by interviews followed by peer debriefing. To verify data credibility, we also considered using the participant validation of the findings, but we were not able to go back to the mothers to validate the findings because of the COVID-19 lockdown restrictions. However, we could return to the healthcare providers to validate the findings of the study during a one-day workshop. To enhance trustworthiness, we also used prompting questions and we carried out triangulation of data sources.

## Results

### Characteristics of the participants

Forty-four respondents, including 12 healthcare providers involved in KMC implementation and 32 mothers who were receiving or received KMC, participated in the study. Regarding healthcare providers’ sample, it was composed of 05 pediatricians, 02 midwives, 02 nurse aids, 02 volunteers and 01 childcare worker. They all had less than 2 years’ experiences in KMC. The sample of healthcare providers comprised 02 males and 10 women and their age varied from 35 to 50 years old. Healthcare providers’ characteristics are described in Table 1.

**Table 1:** Background characteristics of healthcare providers

Interview number	Age (year)	Sexe	Qualifications	years’ experience in KMC (in year)
Interview 1	41	F	pediatrician	1 ─2
Interview 2	43	F	midwife	1 ─2
Interview 3	46	F	midwife	1 ─2
Interview 4	48	F	nurse aid	1 ─2
Interview 5	40	F	nurse aid	1 ─2
Interview 6	39	F	childcare worker	1 ─2
Interview 7	35	F	volunteer staff	1 ─2
Interview 8	36	F	volunteer staff	1 ─2
Interview 9	46	F	pediatrician	1 ─2
Interview 10	44	M	pediatrician	1 ─2
Interview 11	50	M	pediatrician	1 ─2
Interview 12	47	F	pediatrician	1 ─2

Concerning the mothers interviewed, their ages ranged between 18 and 47 years old. Twenty of them were married, living with their husbands and twelve were single/engaged. The number of children varied between 1 and 8. The majority of mothers had attended secondary school, only ten of them had a university level. As for occupation, five of them were students, eleven were housewives, four were civil servants and twelve were self-employed. Mothers’ characteristics are described in Table 2.

**Table 2:** Detailed background characteristics of mothers

	Age (year)	Education level	Occupation	Marital status	Parity
Interview 1	39	University	Civil servant	Married	2
Interview 2	25	Secondary	Housewife	Married	1
Interview 3	34	Secondary	Housewife	Married	3
Interview 4	47	Secondary	Housewife	Married	8
Interview 5	21	Secondary	Housewife	Single/engaged	1
Interview 6	35	Secondary	Self-employed	Married	2
Interview 7	32	Secondary	Self-employed	Married	4
Interview 8	18	High school	Student	Single/engaged	1
Interview 9	28	High school	Housewife	Single/engaged	2
Interview 10	19	University	Student	Single/engaged	1
Interview 11	32	University	Self-employed	Single/engaged	4
Interview 12	40	University	Self-employed	Married	5
Interview 13	30	High school	Self-employed	Married	1
Interview 14	22	Secondary	Self-employed	Single/engaged	2
Interview 15	34	University	Civil servant	Married	3
Interview 16	36	Secondary	Self-employed	Single/engaged	1
Interview 17	25	University	Student	Married	2
Interview 18	29	High school	Housewife	Married	1
Interview 19	30	Secondary	Housewife	Married	3
Interview 20	27	University	Self-employed	Married	1
Interview 21	32	Secondary	Housewife	Married	3
Interview 22	38	Secondary	Self-employed	Married	3
Interview 23	23	University	Student	Single/engaged	1
Interview 24	26	Secondary	Self-employed	Married	2
Interview 25	37	High school	Civil servant	Married	1
Interview 26	31	Primary school	Housewife	Single/engaged	2
Interview 27	23	Secondary	Self-employed	Single/engaged	1
Interview 28	33	Secondary	Housewife	Married	3
Interview 29	20	University	Student	Single/engaged	2
Interview 30	31	Secondary	Housewife	Married	3
Interview 31	33	Secondary	Self-employed	Single/engaged	2
Interview 32	36	University	Civil servant	Married	3

### Identified barriers and facilitators by CFIR domains

Participants described barriers and facilitators of KMC implementation related to the CFIR constructs: intervention characteristics, outer setting, inner setting, characteristics of individuals and process domains. However, the participants did not report barriers or facilitators for every construct. When analyzing and comparing the interviews, in some cases the same theme emerged in both staff and patient interviews, but in other case arose only in staff or patient interviews. Barriers and facilitators are summarized in Table 3.

**Table 3:** Identified barriers and facilitators and their related CFIR construct

CFIR domain	Construct	Barrier	Facilitator
Intervention characteristics	cost of the intervention	Barrier did not emerged from data	Low cost of the intervention
Outer Setting	Patient needs and resources	Lack of community awareness	Facilitator did not emerged from data
		Beliefs about carrying a newborn on the chest	
		Father’s resistance	
	Cosmopolitanism	Barrier did not emerged from data	Partnership and networking with other organizations
Inner setting	Tension for change	Increased workload	Facilitator did not emerged from data
	Leadership engagement	Barrier did not emerged from data	Strong leadership
	Available resources	Lack of food for admitted mothers	Training of healthcare providers
		Lack of space for admission	
		Lack of supplies	
		Insufficiency of human resources	
Characteristics of individuals	Knowledge & Beliefs about the Intervention	Barrier did not emerged from data	Healthcare providers’ perceived value of KMC
			Mothers’adherence to KMC
	Other Personal Attributes	Barrier did not emerged from data	Good relationship between mothers and healthcare providers
Process	Executing	Lack of collaboration between service involved in newborn care	Facilitator did not emerged from data
		Lack of home visits	
		Low rate of exclusive breastfeeding	

#### Intervention characteristics

Under this domain, only one theme identified as a facilitator embedded the construct, the cost of the intervention, which also included costs associated with implementing the intervention such as investment, supply, and opportunity costs.

Indeed, a common driver among mothers was the low cost of the intervention compared to conventional care using incubators, as reported by this mother:

*“It is a practice that does not require too much financial means…my sister has given birth to a premature baby, it is currently in an incubator and it is really expensive” (M20, 27 years old).*

Healthcare providers also agreed with the low cost of the intervention. One healthcare provider explained:

*“In Cote d'Ivoire, a newborn dies every hour due to prematurity because there are not enough incubators. In Abidjan, the capital city with more than 5 million inhabitants, there are less than 50 incubators in public hospitals. For preterm and low birth weight babies, a place in a public hospital is therefore rare. In addition, in the private sector, it is expensive: between 50,000 and 100,000 CFA francs [90 to 180 USD] for a day in an incubator with oxygen. When we know that the minimum salary is 65,000 CFA francs [117 USD], the incubator is not affordable to everyone. At the KMC unit, apart from the food that the patients have to pay for, everything is practically free.” (HP1, pediatrician)*

#### Outer setting

Under this domain, one facilitator (cosmopolitanism) and three barriers (belief about carrying the newborn on the chest, lack of awareness and father’s resistance) were respectively related to the following constructs: cosmopolitanism and patient’s needs and resources.

#### Cosmopolitanism

Cosmopolitanism is the degree to which the KMC unit is externally networked with other external organizations. Four types of organizations emerged as major partners: local health departments such as Programme National de Nutrition, implementing partners/non-governmental organization, associations and other health facilities through the perinatal network. Cosmopolitanism was identified as a facilitator to KMC implementation; as explained by these healthcare providers:

*“With the help of this association, we were able to provide food for mothers for a month” (HP3, midwife).*

*“Through this tripartite partnership we were trained in research methodology, qualitative and quantitative data analysis, scientific article writing and monitoring and evaluation.” (HP2, midwife).*

#### Patient needs and resources

##### Belief about carrying a newborn on the chest

The belief about carrying a newborn on the chest was identified as a barrier. Some healthcare providers reported experience with some mothers who refused to adhere to KMC because carrying a newborn on the chest is not well perceived in their culture.

*"Unfortunately, we are sometimes confronted with mothers who are reluctant to use the method because of their beliefs about carrying the child on their chest…According to these women, in case of danger, if the child is on the chest, it will be the most exposed ". (HP8, volunteer staff)*

##### Lack of community awareness

Another barrier that emerged from interviews was the lack of community awareness. For the participants, this lack of community awareness has led to a lack of information on KMC. Healthcare providers and mothers alike agreed that, as illustrated by the following quotes.

*“Community awareness is a component of this intervention…unfortunately we were not able to realize it... A good sensitization of the community would have made it possible to provide adequate information on KMC and to act on the community's adherence to this practice... ” (HP1, pediatrician)*

In alignment with this statement, one mother declared:

*“ It was only when my child was admitted here that I learned about kangaroo mother care ...I had never heard about it before...that's why the beginning was difficult for me...If it was something I knew before maybe I wouldn't be so worried about my child ... ” (M30, 31 years old)*

##### Father’s resistance

Father’s resistance was also reported by healthcare providers and mothers as a barrier to KMC implementation. Some fathers found it difficult to accept being separated from their spouse and child while the child is not in an incubator. For them, having their wife care for the child without the child being in an incubator means that she can also do so at home. One mother explained this situation as follows:

*“At the beginning, I was doing intermittent Kangaroo because the baby was in the incubator... but since the baby went down to the unit for continuous Kangaroo.... my husband's behavior has changed; for him the fact that the baby is no longer in the incubator means that it is getting better and that we can be discharged…. ” (M7, 32 years old)*

#### Inner setting

Five barriers (increased workload, lack of food for admitted mothers, lack of supplies, lack of space for admission, insufficiency of human resources) and two facilitators (strong leadership, training of healthcare providers) were embedded to following constructs of this domain: tension for change, leadership engagement and available resources.

#### Tension for change

##### Increased workload

For some healthcare providers, one barrier to KMC implementation is the increased workload especially for those working in the other facilities of the perinatal network that refer eligible babies to the KMC unit.

*"At the general hospital of Port-Bouët, I am the only one who has been trained in kangaroo mother care, we have a lot of births here...it is not easy to manage everything. I am in a process of training a nurse aid to help me with this activity.... Given the number of eligible babies that we refer to the KMC unit of CHUT, we are advocating for the opening of a unit in our hospital" (HP11, pediatrician)*

#### Leadership engagement

##### Strong leadership

A common facilitator cited by healthcare providers was the strong leadership of the head of the pediatric service that promotes KMC. The head of the pediatric service ensured provision of training, of equipment through advocacy. As reported by healthcare providers, this strong leadership motivated them to do their best to provide KMC. This statement is illustrated by the following quote.

*“We have started slowly....but look at what level we are today…..we are committed to the implementation of KMC thanks to the leadership of our head of pediatric service who really boosted us and made us adhere to his vision of KMC” (HP1, pediatrician)*

#### Available resources

##### Lack of food for admitted mothers

A common barrier mentioned by healthcare providers and mothers was the lack of food for mothers. The KMC unit did not provide meals to mothers admitted for continuous KMC, which led women to take turns preparing their meals. Moreover, family members were obliged to bring food or money.

*"At this time, the unit does not have a budget to provide food to the mothers. They are therefore obliged to prepare the food themselves in turn… When one of them is cooking, the other mothers or the nurse aids look after her child. We are currently advocating with the national nutrition program and some implementing partners to find a solution to this issue…" (HP1, pediatrician)*

The same concern was expressed by this mother:

*"Hum...when I have to leave my child to go to cook, I am always worried... the times when my sister brings the food, it is still expenses for the transport because we live far away…" (M2, 25 years old)*

##### Lack of space for admission

Another barrier cited by the healthcare provider to KMC implementation was the lack of space for admission bed to receive more preterm newborn that are eligible for KMC, as reported by this healthcare provider:

*"We do not have enough space to hospitalize premature babies eligible for kangaroo mother care. From March 2019 to July 2020, out of 319 newborns hospitalized in neonatology and eligible for SMK, 209 were admitted to the SMK unit. We did not take the other eligible newborns due to lack of beds" (HP2, midwife)*

##### Lack of supplies

The lack of supplies, mainly oxygenator and aspirator, has been reported by some healthcare providers working in the KMC unit. Faced with the lack of oxygenator and aspirator in the KMC unit, they believed that these devices are essential for preterm and LBW babies’ survival and strongly hoped that the unit will get them, as stated by one them:

*"I sincerely wish we could have our own aspirator in the unit. When the babies swallow the wrong way, we go upstairs with them in the NICU to use their aspirator with all the risks that this may entail ". (HP4, nurse aid)*

Another healthcare provider declared:

*"We really need supplies, all the supplies you see here have been provided by an external partner ". (HP3, midwife)*

##### Insufficiency of human resources

For the healthcare providers the insufficiency of human resources impeded KMC implementation. This statement is illustrated by the following quotation:

*" We really need more staff in the unit….Because of the lack of human resources we can’t take our time to do other important activities such as mother’s counselling, which requires time, we are obliged to rush" (HP5, volunteer staff)*

##### Training of healthcare providers

Healthcare providers reported that the training they received was crucial in increasing their knowledge and competences on how the KMC should be provided*.*

*“…We participated in a very interesting training which gave us the knowledge and skills to provide KMC. This training was also a good refresher training for us…..we could go back to the basics such as newborn care with a focus on preterm and low birth weight ” (HP12, pediatrician)*

#### Characteristics of individuals

This domain refers to characteristics of individuals involved in implementation that might influence implementation. Three themes were identified as facilitators were related to the following constructs: knowledge and belief about the intervention and other personal attributes. No barrier was identified under this domain.

#### Knowledge and belief about the intervention

##### Healthcare providers’ perceived value of KMC

Another factor that facilitated the implementation of KMC was the value of the intervention for the healthcare providers. Indeed, the majority of healthcare providers recognized the benefits of this method, which they considered to be a humanization of care, and a form of tasks shifting from the healthcare provider to the mother who became the main actor in the care given to the newborn, as explained by this pediatrician:

*“KMC are a blessing!!!!!....this method will save many premature and low birth weight newborns. I find that KMC are humanized and the mother also participates in these cares which allows her to better assess the evolution of her child than when the child is in an incubator… ” (HP11, pediatrician)*

##### Mothers’ adherence to KMC

The adherence of mothers to KMC was also cited as a facilitator. For the healthcare providers, mothers have understood the value of the intervention which allowed them to adhere to it as stated by this volunteer:

“*The majority of the mothers we received here, recognized the value of this practice for their child...this is what allowed us to carry out our activities without much resistance from them*” (HP7, volunteer staff)

This adherence to SMK is also illustrated by this mother statement:

“*When I first came at the KMC unit...I was afraid to carry my child on my chest and I was not sure that it would help my child develop….but little by little, with what the midwives taught me, I have started to see my child grow up and to have confidence in this method*.” (M15, 34 years)

#### Other personal attributes

Another facilitator that emerged from mothers’ interviews was the good relationship with healthcare providers. Mothers reported feeling supported by healthcare providers at the KMC unit. Mothers considered that the listening skills of healthcare providers, the way they behaved with them and became familiar, facilitated their uptake of KMC, as stated by this mother:

*“At the very beginning I was really anxious, I was afraid to hurt my baby, but the health staff was great, the midwives were patient with me, they supported me and gradually I was able to take care of my child without any problem… ” (M13, 30 years old)*

#### Process

All the themes under this domain were embedded to the construct “executing”. All these themes were identified as barriers to KMC implementation. No facilitator was identified under this domain.

#### Executing

This construct refers to the quality of execution according to what has been planned.

##### Lack of collaboration between services involved in newborn care

A common theme among healthcare providers was the lack of collaboration between services involved in newborn care mainly gynecology services and pediatric services. Healthcare providers agreed that a multidisciplinary collaboration could impact positively preterm and LBW babies’ outcomes. One healthcare provider stated:

"*There is a lack of collaboration between perinatal specialists .... Pediatricians, midwives and obstetricians for instance. The organization of meetings, common staffs between us could really improve outcomes for newborn especially preterm and low birth weight"(HP12, pediatrician)*

##### Lack of home visits

Healthcare providers noted that lack of human resources made it difficult for the unit to conduct home visits after the discharge of the dyad. They recognized the importance of home visits to continue KMC within the community as noted by one of them:

*"Unfortunately, we do not yet make home visits, which does not allow us to appreciate the continuity of KMC within the community"* (HP1, pediatrician)

##### Low rate of exclusive breastfeeding

Exclusive breastfeeding is one of the components of KMC, but few mothers were conducting exclusive breastfeeding. This was considered as a hindrance to KMC implementation by some healthcare providers, as stated by one of them:

*“Normally KMC is supposed to increase exclusive breastfeeding, but here when you look at the numbers the breastfeeding rate is low, mixed feeding is what women do most....we really need to address this …. ” (HP4, nurse aid)*

### Proposed solutions to improve KMC implementation

The healthcare providers were asked about suggestions to improve KMC implementation at the unit. The proposed solutions were volunteer staff motivation, mothers and families education and counselling, the recruitment of a psychologist, involvement of all stakeholders.

#### Volunteer staff motivation

One solution to improve KMC implementation and service delivery was to motivate the volunteer staff through financial incentive.

*“Volunteer staff is a great help in the implementation of SMK given the shortage staff and they need to be motivated. As far as I’m concerned their participation in training is not enough to motivate. If we want to retain this staff we need to give them financial incentives.... ” (HP3, midwife).*

#### Intensifying mothers and families education and counselling

According to the majority of healthcare providers, there is a need to intensify education and counselling of mothers and families on KMC by the use of appropriate messages to make them totally adhere to the intervention.

*“ I think that we need more time, more information and education tools to obtain the adherence of mothers and their relatives.... ” (HP5, nurse aid)*

#### The recruitment of a psychologist

For the majority of healthcare providers interviewed, the presence of a psychologist in the unit could help support mothers during their admissions especially young mothers as they report more psychological distress.

*“ We need a psychologist in the unit ....we have had cases of young mothers who could not practice the method because it was their first pregnancy with a preterm newborn. Most of these young mothers do not necessarily have the support of their families or the child's father, which puts them in a stressful situation that does not facilitate the production of milk.”* (HP2, midwife)

#### Involvement of all stakeholders

A common proposed solution was the involvement of all stakeholders to improve KMC implementation. For this healthcare provider besides the user engagement, the success of the implementation also requires the involvement of stakeholders from diverse background notably community, policy makers, the technical structures of the ministry of health, the partners of implementation, private structures, technical and financial partners.

*“ For KMC implementation to be successful, we need the commitment of all stakeholders. For example, structures such as the National Nutrition Program and the World Food Program can help us provide food to mothers in a sustainable way.”* (HP11, pediatrician)

## Discussion

This is the first study carried out in Cote d’Ivoire to identify barriers and facilitators to KMC implementation in the first KMC unit at the CHUT, as well as proposed solutions to improve provision and uptake of KMC. The identified barriers and facilitators influencing KMC implementation were mapped to the CFIR framework. For the scaling up of KMC, it is fundamental to take these barriers and facilitators into account in order to design and adopt implementation strategies for a better rolling out of the intervention.

### Barriers of KMC implementation

Eleven themes emerged as barriers to KMC implementation. Regarding the lack of food for admitted mothers, the lack of space for admission, the lack of supplies, the insufficiency of human resources and increased workload, evidence from other countries showed that the above mentioned barriers were important factors influencing the adoption of KMC [5, 22, 25–28, 36, 38, 40, 41]. For instance, in a study conducted in Ghana, food was often unavailable at facilities for mothers, therefore mothers were unable to breastfeed their newborns, left the hospital early, or depended on relatives for food [41]. Concerning, the lack of space, similar to our findings, a study in Uganda found that intermittent skin-to-skin care was practiced instead of continuous care due to lack of suitable environments in 75% of cases, including lack of beds and space [36]. One consequence of the lack of space for admission is that many babies that are eligible for KMC cannot benefit from the intervention. To overcome this hindrance, an advocacy led by the KMC unit is ongoing to obtain the construction of a larger building with a capacity of 40 beds within the CHUT.

In addition to the lack of space, staff shortage also leads mothers to practice intermittent rather than continuous KMC. One study reported a discharge in under two hours or restricting visitation policies because of staffing shortages and space concerns [41]. One consequence of the staffing shortage is increased workload. In a study in Malawi, in certain facilities when there was an overabundance of patients, KMC mothers were the lowest priority for healthcare providers [15]. However, in other cases, healthcare providers did not believe that KMC increased their workload, or decreased their time spent on other infants [25]. Furthermore, it has also been shown that after an initial increase in workload at the start of KMC implementation reduces nurse’s workload [15].

Effective implementation of KMC also needs available supplies, in our study the majority of the supplies were not provided by the hospital management but external partners. Evidence from other studies have also highlighted the lack of supplies as a major hindrance [22, 25–27, 40]. In a study in Uganda, each of the assessed health facilities reported the provision of equipment and materials by private and external partners [13].

Concerning cultural barriers, the belief that babies should be carried on the back not on the front has been frequently mentioned in many studies [13, 15, 27, 29, 33, 35, 41]. In studies conducted in Ghana and Malawi, it seemed strange to place the baby on the front as instructed by KMC [15, 41]. In another study carried out in Indonesia, mothers felt that skin to skin contact hurt and made infants uncomfortable [29].

Regarding family support that includes approval of fathers/husbands, it was identified as a factor facilitating the implementation of KMC in a review on Sub Saharan countries [27]. However, in our study, we found that father’s resistance to the practice of KMC was a major concern. Father’s resistance meant that they could not conceive having their wife care for the child without the child being in an incubator, meaning that she could also do so at home. This barrier seems to be unique to the country. In a systematic review that also sought to identify the most frequently reported barriers to KMC practice for fathers, the indexed of the barriers from the most to the less reported were: lack of opportunity to practice, issues related to gender role, lack of help with KMC practice and other obligation, fear/anxiety to hurt the child, pain/fatigue, issues with facilities environment and resources, positioning issue, issues with clothing/medical devices [26]. A study from Ethiopia revealed that male dominance in a patriarchal society may override the mother’s acceptance and practice of KMC, as duration of hospital stay may be often determined by the father’s decision-making [30].

Additionally, similar to previous studies, lack of community awareness has been reported as a major bottleneck to KMC implementation [25–27, 30]. As mother acceptance and practice of KMC is inextricably linked with community acceptance [30], there is therefore a need to strengthen this weakness and create awareness at community level. Facility-based KMC services should incorporate a community sensitization component within their activities and explore innovative ways to link with community-based health initiatives [27].

As regards the process of implementation, a major hindrance was the lack of home visits by the medical staff to follow up the dyad within the community. Evidence from others studies also pointed out the influence of lack of follow up on KMC implementation [25, 33]. Health system’s capacity to follow up preterm infants after discharge is probably the greatest challenge for LMICs [33]. Moreover, the availability of material and human resources play an important role in the follow up of the dyad after discharge by the staff of the health facility [25].

As for the lack of collaboration between services in charge of newborn care, multidisciplinary meetings should be promoted and organized at hospital level. In a study conducted in the United States, weekly multidisciplinary meetings to coordinate and provide continuity of care for medically complex neonates in a NICU was associated with improved patient outcomes [45].

Concerning exclusive breastfeeding, despite the fact that healthcare providers have been trained in breastfeeding management and counselling, the low rate of exclusive breastfeeding among mothers admitted in the KMC unit is impeding KMC implementation. Similar findings have been reported in a study conducted in Indonesia, where none of LBWI’s mothers exclusively breastfed, the infants have been given formula milk [29]. Exclusive breastfeeding is a central pillar of KMC [4] not only for its nutritional benefits, but also for the connection that it builds. However, in the current context of CHUT, where the maternity ward is undergoing rehabilitation, the majority of children are referred from other health facilities and early breastfeeding is not always done at birth. The delay in transfer and the stress of hospitalization could partly explain this low rate of exclusive breastfeeding. However, in a systematic review, where different conceptualizations of what comprises KMC emerged, two-thirds of the studies were ambiguous on the onset of KMC practice; and supporting breastfeeding appeared to be a potentially neglected area.

Therefore it is important for the medical staff of the KMC unit to educate and encourage mother to adopt exclusive breastfeeding. Moreover, health facilities that refer newborn to the unit should initiate early breastfeeding and skin to skin contact during transfer. On the other hand, refresher trainings on breastfeeding management and counselling should also be planned.

Among all the above-mentioned barriers, the lack of collaboration between services in charge of newborn care and father’s resistance seems to be specific to Cote d’Ivoire.

### Facilitators of KMC implementation

As for facilitators of KMC implementation, seven themes emerged from our findings. The low cost of the intervention has been identified as a potential facilitator in many studies [24, 27]. However, in some countries the indirect and direct cost of the intervention are major financial barriers. In a study conducted in Ethiopia, most participants revealed that long distances to KMC hospitals, high cost for transportation payment for bed services, catering services, and medication expenses, and inability to buy KMC clothes were major financial barriers to KMC uptake [30]. In Nigeria, the need was mentioned to lower hospital costs to families to support an extended stay during KMC [32].

In addition, in many other settings as in Cote d’Ivoire, cosmopolitanism is a key factor facilitating KMC implementation through networking and partnership with other organizations. Through partnership and networking, the KMC unit was able to obtain supplies, training sessions as well as food for admitted mothers. A study from Uganda also showed that training, supportive supervision and resources were provided by external partners [13].

As in many studies, training of healthcare providers has been identified as a potential enabler of KMC implementation [27]. Effective training methods were pre-service curricula in nursing and medical programs, complemented by continuous in-service training, face-to-face facilitation with multimedia materials and training sessions, coordinated by regional levels and refreshed by meetings, workshops and exposure to current literature on the topic [27]. Effective adoption of KMC also requires a leadership within the organization [13, 33, 38]. Even if there is a strong leadership within the unit; it has to face hospital management for which KMC is not seen as a hospital priority and limited resources are allocated to the unit. Therefore, the unit only operates on donations offered by external partners. A study in Uganda highlighted how the presence of the hospital director at all meetings strengthened efforts and support of senior management allowed for staff to be trained and space alterations [13]. In another study in Indonesia, the commitment to KMC at the hospital management level was strongly perceived as a supporting factor [40].

Acceptance and good perception and attitudes of the main actors of KMC implementation, healthcare providers and mothers; is an enabler of KMC provision and uptake as demonstrated in other studies [23–25, 27, 40]. Moreover the good interpersonal relationship between healthcare providers and mothers, with staff supporting the mothers, was also identified as an enabler of KMC adoption in LMIC [26].

### Proposed solutions to improve KMC implementation

To improve the implementation of KMC, healthcare providers have proposed some solutions such as the motivation of the volunteer staff, intensifying mothers and family education and counseling, the involvement of all stakeholders, the recruitment of a psychologist.

Regarding volunteer staff, even though to be a volunteer is to participate willingly in the life of the association without financial compensation, volunteers usually contribute to the greater good as long as they feel they are making a meaningful contribution [46]. However, in a context where there is an insufficiency in human resources, motivate volunteer staff could help to retain them and ensure the continuity of care. The challenge is to find the best way to motivate them. Even though in our study, healthcare providers proposed to give financial incentives, some studies demonstrated that the amount given may impact the work of the volunteer. Indeed, a study showed that giving small money to volunteers did not help and impacted negatively their work. On the other hand, when volunteers in the same experiment were offered larger monetary rewards, the hours they offered to work were high; showing that in the absence of budget constraints, money may help to motivate volunteer staff [46, 47]. Another study indicated that a volunteer program could meet needs and be a motivational force for both individual volunteer and the organization. However, it requires coordination and some secure funding to remain sustainable [48].

Concerning the involvement of all stakeholders, the support from family members was documented in many studies and emerged as the top enabler of practice in sub-Saharan Africa [27]. Besides family support, government, implementing partners, professional association such as pediatrics society should be involved in the rolling out of KMC. However, one issue with external partners which provide supplies, is that the quality of care may deteriorate as projects end. Constant engagement from government, health workers through supportive supervision, mentorship and knowledge sharing of best practices and local leadership is required to sustain the project [27].

In terms of intensifying education and counselling for mothers and families education on KMC, it is very important to devote more time on these activities. As KMC seems for some communities an unusual way of carrying the baby, it is fundamental for them to comprehend what it entails, as well as its benefits. In a study carried out in Zambia, mothers accepted KMC after they were given information and education through good communication [23]. Continuing education of medical staff and parents is necessary. Moreover, providing a better liaison with antenatal care services to endorse KMC could contribute to better preparation of caregivers and training of healthcare providers [22]. Besides community awareness and education by healthcare providers, peer support from other mothers or fathers through sharing their kangaroo mother care experiences could help promote household acceptance.

The recruitment of a psychologist in the unit was also proposed as a solution to improve KMC implementation. The presence of a psychologist could be important for the support of the mother and the family members because although it was found that KMC can promote more positive parent-child interaction, it can also improve negative maternal mood such as anxiety or depression [49].

This study presented interesting results, but those results were limited by some factors. For instance, we acknowledged the possibility of social *desirability bias* from mothers when they participated in the interview. In order to minimize this bias, we conducted interviews with mothers in a separate room and we used non-hospital staff as research members. We could present and validate the study findings with healthcare providers during a workshop. However, we were not able to return to mothers to validate the study findings and this is also acknowledged as a limitation. Another limitation is the fact that we could not carry out method triangulation by conducting focus group in the community. Further studies within the community will help to understand the challenges in practicing KMC outside the hospital.

## Conclusion

KMC is a complex intervention and understand the context specific barriers and facilitators to its implementation allows the scaling-up of KMC to be tailored to the population and healthcare providers’ needs. Our study results revealed some unique barriers to KMC such as the lack of collaboration between services in charge of newborn and father’s resistance. The study also allows proposing solutions to improve KMC implementation. To overcome challenges to implement KMC, it is crucial to strengthen health system, community support and link between community level and the hospital level services.

## Supplementary Information

**Additional file 1.** Interview guides for mothers and healthcare providers.

## Data Availability

The data presented in this study are from the Project « *Documentation of Kangaroo Mother Care Implementation in the Neonatal Intensive Care Unit of the Pediatric Services of the Teaching Hospital of Treichville »* conducted in Cote d’Ivoire. Access will be granted from the corresponding author on reasonable request, only after careful and due consideration of the compliance with the ethics requirements, the data policy of the Cellule de Recherche en Santé de la Reproduction»***.***

## Abbreviations

CHUT: Teaching hospital of Treichville
HP: Healthcare provider
KMC: Kangaroo Mother Care
LMIC: Low and middle income countries
M: mothers
NICU: Neonatal Intensive Care Unit
WHO: World Health Organization
