# Supplementary material for: Barriers and facilitators to kangaroo mother care implementation in Cote d’Ivoire: a qualitative study
Source: BMC Health Serv Res. 2021 Nov 9;21:1211. doi: 10.1186/s12913-021-07086-9 (PMC8576306; doi:10.1186/s12913-021-07086-9)
Supplement: Supplementary file 1 — Additional file 1. Interview guides for mothers and healthcare providers. [file 12913_2021_7086_MOESM1_ESM.docx]

**Interview guide for mothers**

| Practice of KMC | - Did you know about KMC before your admission? When and where did you first hear about KMC? - Was anyone in your family aware of KMC before your admission? - What do you and your family think about SMK? Can you tell us what the benefits are for the mother, baby and family of performing KMC? Who took the decision of the admission into your household - What do other mothers and their family think about SMK? - How do you perform KMC? –Probes : procedures, materiel, relationship with healthcare providers, Covid19 etc) - Does the hospital provide adequate resources (e.g. bed, clothes, bandage, glass, cups) for you to perform KMC? Probe (baby wrap, food, bed, armchair, etc) - If not what are the resources that you need and how do get them? - Can you describe your relationship with the medical staff, how do you cooperate them? What did you think of the health providers in the facility in terms of attitude towards you and your baby? - Do you feel that dyads needs and resources are a priority for the unit - Do you encounter any difficulty; what were these |
| --- | --- |
| Barriers and facilitators | - According to you what were the barriers to KMC adoption and practice - According to you what were the factors that facilitated KMC adoption and practice a |
| 5/ Suggestions (proposed solutions to improve KMC) | - Is there anything else you would like to discuss that has not been addressed? |

End

Thanks for your participation

**Interview guide for healthcare providers**

| Practice of KMC | - What do you know on KMC? - When was the last time you received a refresher training on KMC. Probe: What the training was about?   Do you think that you need more training?   - What do you think of KMC in the management of preterm low birth weight infants?   Probe: How do your colleagues see it?   - How is KMC organized? (probe: equipment; materials and human resources, Covid19) - Do have think your workload has increased since the opening of the unit? Explain - How do you collaborate with the other health facilities that refer newborn at the KMC unit?   If the healthcare provider works at a health facility of the perinatal network ask how do they collaborate with   - What do you think about mothers ‘acceptance of KMC? - What is the family and community support during KMC? - Do you think that infrastructure of the unit affect the implementation of the KMC? - Do you think the current resource is adequate for KMC?   Probe: How to acquire those resources? What’s the challenge?   - How does the hospital support the unit to implement KMC?   Have you received any resource from hospital? What resource is easier to get from the hospital?   - Have you any collaboration with other organization   Probes : How are they involved in KMC implementation   - Are there any activities planned that you could not carried out? What were the reasons? - Is there any leadership, champion within the unit to promote KMC? |
| --- | --- |
| Barriers and facilitators | - According to you what are the factors impeding KMC implementation? - According to you what are the factors facilitating KMC implementation? |
| 5/ Suggestions (proposed solutions to improve KMC) | What solutions do you propose to improve the implementation of kangaroo mother care?   - Is there anything else you would like to discuss that has not been addressed? |

End

Thanks for your participation
